# Supplementary material for: Determination of SLES in Personal Care Products by Colloid Titration with Light Reflection Measurements
Source: Molecules. 2021 May 5;26(9):2716. doi: 10.3390/molecules26092716 (PMC8124727; doi:10.3390/molecules26092716)
Supplement: Supplementary file 1 [file molecules-26-02716-s001.zip › Fig.S1 - szampony-fot-4f.pdf]

Determination of SLES in personal care products by colloid titration with light reflection measurements

Dorota Ziółkowska, Iryna Syrotynska, Alexander Shyichuk and Jan Lamkiewicz

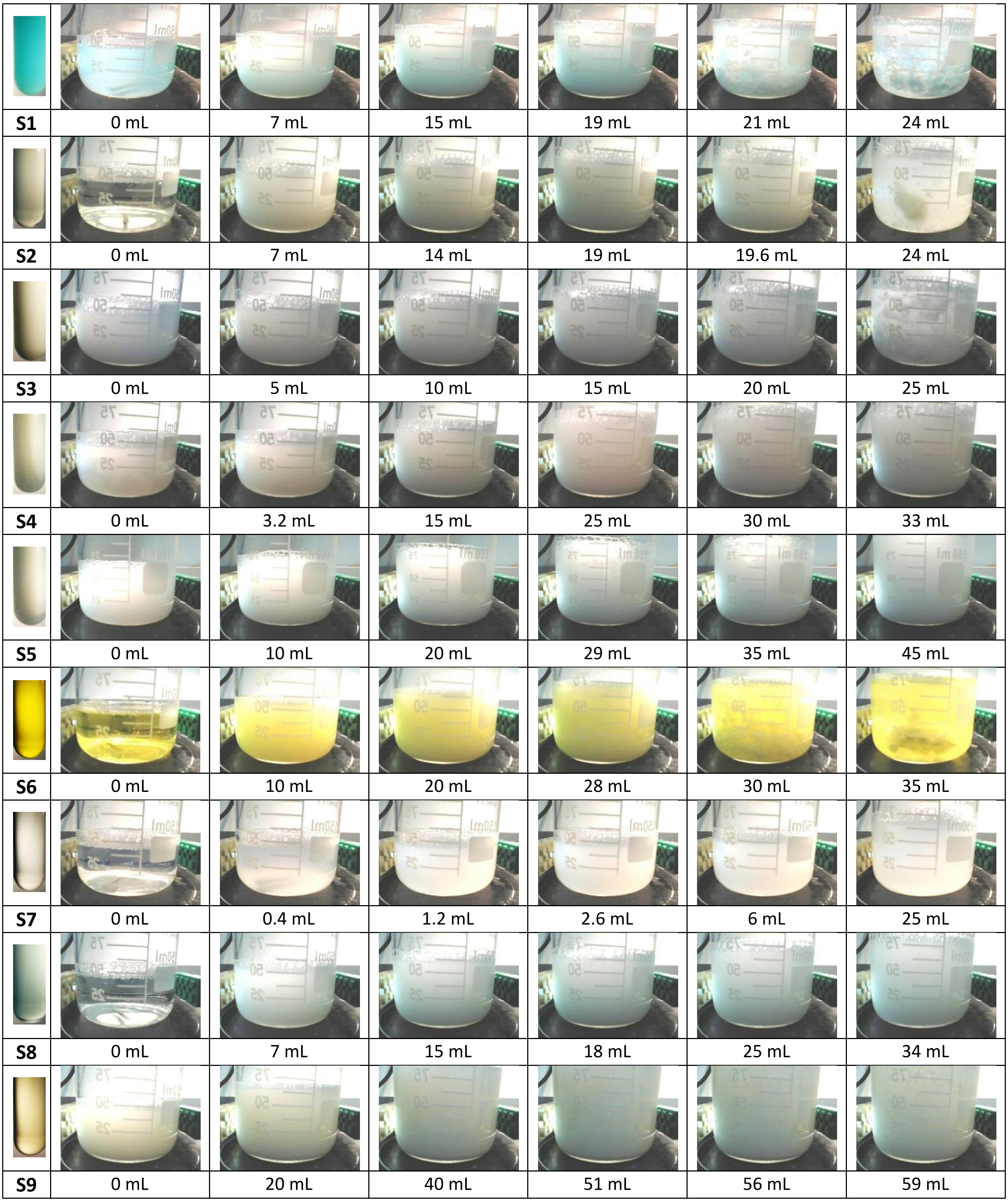

Figure S1. Photographs of analysed products as well as product-polyDADMAC mixtures at indicated volumes of polyDADMAC added
